# A common missense variant in *BRCA2 *predisposes to early onset breast cancer

**DOI:** 10.1186/bcr1338

**Published:** 2005-10-24

**Authors:** Bohdan Górski, Steven A Narod, Jan Lubiński

**Affiliations:** 1 Department of Genetics and Pathology, International Hereditary Cancer Center, Pomeranian Medical University, Szczecin, Poland; 2 Centre for Research on Women's Health, University of Toronto, Canada

## Abstract

**Introduction:**

Mutations in the *BRCA2 *gene are one of the two major causes of hereditary breast cancer. Protein-truncating mutations of BRCA2 are usually deleterious and increase the risk of breast cancer up to 80% over a lifetime. A few missense mutations in *BRCA2 *are believed to have a similarly high penetrance, apart from more common neutral polymorphisms. It is often difficult to classify a particular sequence variant as a mutation or a polymorphism. For a deleterious variant, one would expect a greater allele frequency in breast cancer cases than in ethnic-matched controls. In contrast, neutral polymorphic variants should be equally frequent in the two groups.

**Methods:**

We genotyped 3,241 cases of breast cancer diagnosed at under 51 years of age, unselected for family history, from 18 hospitals throughout Poland and 2,791 ethnic-matched controls for a single *BRCA2 *C5972T variant.

**Results:**

The variant was present in approximately 6% of the Polish population. In the study, 13 women (11 cases and two controls (OR = 4.7; p = 0.02)) were homozygous for the variant allele. The overall odds ratio for breast cancer in women with a single copy of the *BRCA2 *C5972T variant was 1.1 (p = 0.7); however, the effect was significant for patients diagnosed at or before age 40 (OR = 1.4; p = 0.04). We reviewed the association between the *BRCA2 *variant in different histologic subgroups and found the effect most pronounced in women who had ductal carcinoma *in situ *(DCIS) with micro-invasion (OR = 2.8; p < 0.0001).

**Conclusion:**

The *BRCA2 *C5972T allele is a common variant in Poland that increases the risk of DCIS with micro-invasion. The homozygous state is rare but increases the risk of breast cancer five-fold.

## Introduction

There are several approaches to identifying low-penetrance candidate genes for breast cancer. In one approach, it is assumed that missense variants of genes for which truncating mutations are clearly pathogenic might also be deleterious. If the missense allele demonstrates high penetrance (i.e. like truncating mutations), then it will be relatively straightforward to establish the association when allele frequency is high. If the penetrance of the missense variant is low, however, then the association may be missed if only a small number of cancers is studied and the variant may falsely be classified as a neutral polymorphism. We are in the process of establishing a large database of breast cancer cases and ethnic-matched controls in order to evaluate the pathogenicity of the common founder alleles of the most important cancer susceptibility genes. Several deleterious founder alleles have been identified in *BRCA1*, but to date, no founder mutation in *BRCA2 *has been identified [1-3]. There are a few common variant alleles in *BRCA2 *in Poland; one of these (C5972T) changes the amino acid sequence of BRCA2 from threonine to methionine at codon 1915. It lies within the range of the BRC encoded by exon 11 that are thought to be involved in binding to RAD51 [4]. We sought to determine whether this common missense BRCA2 variant plays a role in breast cancer susceptibility.

## Materials and methods

### Study subjects

The study population included prospectively ascertained cases of invasive breast cancer diagnosed at 50 years of age or less at 18 treatment centers throughout Poland between 1997 and 2003. Invitation to participate was delivered during hospital stay or by mail. During the interview, the goals of the study were explained, informed consent was obtained, genetic counseling was offered, and a blood sample was taken for DNA analysis. A detailed family history of cancer was taken (first-, second-, and third-degree relatives included) and a risk factor questionnaire was completed. A total of 4,778 cases of early onset invasive breast cancer were identified at the 18 participating centers during the study period. Of these, 3,627 women (76%) accepted to participate and provided a blood sample for DNA analyses, 114 died, and 16 refused to participate. The medical record and pathology report were reviewed. In the end, we had DNA available for genotyping from 3,241 patients.

### Controls

Two control groups were combined. The first group consisted of 2,000 unselected neonates from ten hospitals throughout Poland born in 2003 and 2004. Samples of cord blood were forwarded to the study center in Szczecin. The second control group consisted of 1,000 adults from the region of Szczecin unselected for cancer family history, sex, or age. We genotyped 2,791 controls for the C5972T allele, 1,993 neonates and 798 adults. To ensure comparability of the control groups, C5729T allele frequencies were computed separately for the adult and neonatal control groups and compared. The study protocol was approved by the ethics committee of the Pomeranian Medical University.

### Laboratory methods

All experiments were performed at the Department of Genetics and Pathology, Pomeranian Medical University, Szczecin, Poland.

#### DNA isolation

DNA samples were obtained from peripheral blood of adults or from cord blood of neonates using the non-enzymatic rapid method as described previously [5].

#### Analysis of the C5972T variant

The C5972T variant (Thr1915Met) was analyzed by restriction fragment length polymorphism PCR using b5972F (5'-CTC TCT AGA TAA TGA TGA ATG ATG CA) and b5972R (5'-CCA AAC TAA CAT CAC AAG GTG) primers. The forward primer introduces an artificial restriction site for the Mph1103I enzyme (Fermentas, St. Leon-Rot, Germany)

PCR products were digested in mutation positive cases. PCR products were separated in 3% agarose gel and visualized in ultraviolet light. For positive cases with RFLP-PCR (cleaved by Mph1103I), a DNA sample was sequenced to confirm the presence of the mutation. Additionally, 72 randomly selected samples negative with RFLP-PCR for the C5972T variant were sequenced. The results of RFLP-PCR and of direct DNA sequencing were 100% concordant.

### DNA sequencing

A fragment of *BRCA2 *exon 11 was amplified by PCR using b5972F/b5972R primers. The purified PCR products were sequenced directly with a BigDye Terminator v3.0 DNA Sequencing Kit (Perkin Elmer, Foster City, CA, USA) and the same reverse primer used previously for the amplification of exon 11 of the *BRCA2 *gene. Products of sequencing reactions were separated and analysed on the ABI 377 DNA Sequencer (Perkin Elmer).

### *BRCA1 *testing

All breast cancer patients were tested as described previously [4] for three mutations (5382insC, C61G, 4153delA) representing 80% to 90% of all high-risk *BRCA1 *changes in Poland.

### Statistical analysis

Statistical analysis included a comparison of the prevalence of the C5972T allele in cases and controls. Odds ratios (ORs) were generated from two-by-two tables and statistical significance was assessed with the chi-square test. *BRCA2 *nucleotides were identified according to the GeneBank U43746 sequence.

## Results

The C5972T variant was detected in 8.3% of unselected breast cancer patients diagnosed at age 40 years or below, in 5.7% of cases diagnosed from age 41 to 50 years, and in 5.8% of Polish controls. The overall OR for breast cancer with a C5972T mutation was 1.1 (95% CI: 0.8–1.3); however, in women less than 40 years old the OR was 1.4 (95% CI: 1.0 – 1.9) (Table 1).

We found 13 C5972T homozygotes (11 patients and 2 controls). The OR associated with the homozygous state was 4.7 (95% CI: 1.1 to 21.4). Among 11 cases of breast cancer diagnosed in homozygotes, 10 were diagnosed between ages 40 and 50 years. The 11 homozygotes had predominantly intraductal (ductal carcinoma *in situ *(DCIS) with micro-invasion, 4 cases), ductal G3 (4 cases), lobular (2 cases), and ductal G2 (1 case) cancers. None of the first- or second-degree relatives of the homozygotes were diagnosed with breast or ovarian cancer.

We then explored the characteristics of the breast cancers that were identified in women with the *BRCA2 *C5972T allele (Table 2) and compared them with tumors in women without the predisposing allele (women with *BRCA1 *mutations were excluded from all comparisons and women who received pre-operative chemotherapy were excluded from the histological comparisons). Women with the *BRCA2 *variant were more likely to have bilateral cancer (5% versus 3%) or multifocal cancer (30% versus 23%) but these differences were not statistically significant. However, a significant effect was observed with intraductal cancer. Among the 5972C/T heterozygotes, 18 of 103 tumors were predominantly intraductal (17%), compared to 7% in 5972C/C breast cancer patients (OR = 2.82; p ≤ 0.0001; 95% CI: 1.6–4.8). A heterozygous *BRCA2 *variant was present in 13% of the women with predominantly intraductal cancer. Compared to the population controls, the OR for intraductal cancer given a (heterozygous) form of *BRCA2 *variant was 2.5 (p = 0.0005; 95% CI: 1.5–4.1). For women under 40 years old, the association was stronger (OR = 4.1; p = 0.006; 95% CI: 1.2–8.3).

## Discussion

The C5972T allele of the *BRCA2 *gene appears to be over-represented in women with early onset breast cancer in Poland compared to controls. Although the effect was modest in the overall data set, it was stronger for women who had predominantly intraductal breast cancer and for women who were homozygous for the variant allele. Existing reports on the C5972T variant do not record any case of homozygote TT genotypes [6,7]. It is of interest that the heterozygote state was associated with early onset breast cancer and the homozygote state with later onset cancer (above 40 years of age). To our knowledge, this has not been seen before and there is no compelling explanation; however, we believe that the sizes of these subgroups are small and require confirmation in other studies.

Our study population consisted of DCIS cases with a micro-invasive component only. To our knowledge, this is the first genetic association reported for intraductal cancer with micro-invasion. Our data set did not include cases of DCIS alone and we were unable to assess the effect of the *BRCA2 *C5972T allele in women with predominantly invasive cancer and associated DCIS. These topics will be the focus of future studies. We did not see other pathological features that distinguished *BRCA2*-associated tumors from tumors in non-carriers. This situation is not different from that described for *BRCA2 *carriers in general [8-10].

Several previous studies have shown that DCIS is a characteristic feature of cancers in *BRCA2 *carriers [11-14]. In the case of *BRCA2*-associated breast cancers, it appears that loss of heterozygosity (LOH) occurs as a means of silencing the second allele. In one family with a germline *BRCA2 *mutation, three cases of invasive breast cancer and one case of DCIS were observed [13]. LOH at the *BRCA2 *locus was demonstrated in all four cases. The authors suggest that DCIS and LOH in the *BRCA2 *locus may be common early steps in the development of invasive breast cancer in *BRCA2 *carriers. The LOH data support the concept that *BRCA2 *behaves as a tumor suppressor gene. In support of this hypothesis are our observations of a greater breast cancer risk in women with two inherited 5972T variants and a lower frequency of positive family history among 5972C/T heterozygotes.

In our study, we observed no association between the examined variant and increased family history. This may be explained by the modest association of the examined variant with breast cancer risk and probable recessive pattern of inheritance (the highest OR values were observed in homozygotes, all of them with negative breast cancer family history).

Actually, at least some of the 5972C/T heterozygotes may be compound heterozygotes, with the second allele being another *BRCA2 *variant.

We now provide epidemiological and clinical evidence demonstrating the deleterious nature of the C5972T allele. To our knowledge, functional studies have not been performed on this variant. Few other deleterious missense variants in *BRCA2 *have been identified.

The HH genotype of the non-conservative amino acid substitution polymorphism N372H in the *BRCA2 *gene was reported to be associated with a 1.3-fold to 1.5-fold increase in the risk of both breast and ovarian cancer [15-18], but there have been negative studies as well [19,20].

Our control group was drawn both from the newborns of 10 Polish cities and from the adult population in the region of Szczecin; however, the frequency of alleles was similar in the newborn (5.7%) and adult (5.8%) populations. There was no evidence that the genotype frequencies of the C5972T variants deviated from those expected from the Hardy-Weinberg equilibrium for the combined control group (p = 0.79) or for newborn and adult control groups (p = 0.59 and p = 0.73). There was no statistical difference in the C5972T allele frequencies in newborns recruited from the Szczecin metropolitan region compared to other Polish cities (data not shown). According to existing literature data, the frequency of the C5972T variant is similar in other populations such as North American, Austrian and Oceanian [6,7].

## Conclusion

The heterozygosity of the *BRCA2 *C5972T variant appears to be a neutral polymorphism in women above the age of 40 but may be deleterious prior to age 40. The risk associated with this allele was modest (OR = 1.4) in young women. Strong associations were not observed until cases were subclassified by their histological appearance or by homozygous state. It is important to confirm these observations in other populations. Large, well-controlled studies are needed to establish the full range of risks associated with other *BRCA2 *alleles before they can definitely be identified as neutral. Identification of breast cancer susceptibility genes that are associated with modest penetrance requires very large association studies, unless the high-risk alleles are very common.

## Abbreviations

DCIS = ductal carcinoma *in situ*; LOH = loss of heterozygosity; OR = odds ratio; RFLP-PCR = restriction fragment length polymorphism polymerase chain reaction.

## Competing interests

The authors declare that they have no competing interests.

## Authors' contributions

BG designed and performed lab assays. BG and JL conducted data analysis and drafted the manuscript. BG, JL and SN contributed to interpretation of results and revision of the manuscript.

## Figures and Tables

**Table 1:** Frequencies of the *BRCA2 *C5972T variant allele in breast cancer patients and controls

Variant	Controls	Cases	Odds ratio	p-value	95% CI
All subjects					
CC	2,630	3,039	1.0		
CT	159	191	1.0	0.74	[0.8–1.3 ]
TT	2	11	4.8	0.02	[1.1–21.4]
CT+TT	161	202	1.1	0.45	[0.9–1.3 ]
Total	2,791	3,241			
					
Cases under age 40 years					
CC	2,630	598	1.0		
CT	159	51	1.4	0.04	[1.0–2.0 ]
TT	2	1	2.2	0.46	[0.2–23.7]
CT+TT	161	52	1.4	0.03	[1.0–1.9 ]
Total	2,791	650			
					
Cases 41+ years					
CC	2,630	2,441	1.0		
CT	159	140	0.9	0.68	[0.8–1.2 ]
TT	2	10	5.4	0.02	[1.2–24.6]
CT+TT	161	150	1.0	0.97	[0.8–1.3 ]
Total	2,791	2,591			

CI, confidence interval.

**Table 2:** Characteristics of breast cancers associated with 5972C/T or TT variants and wild-type *BRCA2 *sequence

	5972C/T or TT		Wild type		OR^a^	p-value	95% CI
	N	%	N	%			
Histology							
Medullary	7	6	122	7	0.86	0.71	[0.4–1.9]
Ductal	41	36	607	35	1.04	0.83	[0.7–1.5]
Tubular or tubulo-lobular	3	3	104	6	0.42	0.13	[0.1–1.3]
Lobular	22	19	364	21	0.90	0.70	[0.5–1.5]
DCIS+micro-invasion	22	19	121	7	3.18	<0.0001	[1.9–5.2]
Other	19	17	416	24	0.63	0.09	[0.4–1.0]
Multifocal	19	30	211	23	1.47	0.17	[0.8–2.6]
Bilateral	7	5	63	3	1.71	0.18	[0.7–3.8]
ER+	50	67	586	62	1.24	0.40	[0.7–2.0]
Family history+^b^							
All patients	32	21	569	26	0.72	0.14	[0.5–1.2]
Diagnosed at <40 years	4	10	97	24	0.36	0.06	[0.1–1.1]
							
	0		0		0	p-value	95% CI
Mean age (years)^c^	43.9		44.4		0.5	0.34	[-0.5–1.5]
Mean tumor size (cm)^c^	2.0		2.1		0.1	0.41	[-0.1–0.3]

^a^Odds ratios (ORs) for individual variables represent the odds of being a mutation carrier for a woman with a particular characteristic, compared to all other women who did not have the characteristic. For histology the comparisons were made for one histological group to all other histologies. ^b^Family history+ indicates the occurrence of one or more breast or ovarian cancers among first and second degree relatives. ^c^For age and tumor size comparisons, p-values and confidence intervals refer to the difference of means. Data were missing from the following categories and these cases were excluded from the specific calculations: histology (909 cases), multi-focality (1,774 cases); bilaterality (744 cases), estrogen receptor status (1,746 cases), family history (756 cases), family history in group diagnosed <40 (150 cases). Patients who received neo-adjuvant chemotherapy were excluded from all analyses with the exception of family history. 184 patients with *BRCA1* mutations were excluded from all analyses. ER+, estrogen receptor expression positive.
